# Preparing for Ebola Virus Disease in West African countries not yet affected: perspectives from Ghanaian health professionals

**DOI:** 10.1186/s12992-015-0094-z

**Published:** 2015-02-26

**Authors:** Yaw Nyarko, Lewis Goldfrank, Gbenga Ogedegbe, Sari Soghoian, Ama de-Graft Aikins, NYU-UG-KBTH Ghana Ebola Working Group

**Affiliations:** Centre for Technology and Economic Development, New York University, New York, USA; Department of Emergency Medicine, New York University, New York, USA; Global Institute of Public Health, New York University, New York, USA; Korle Bu Teaching Hospital, Accra, Ghana; Centre for Social Policy Studies, University of Ghana, Accra, Ghana

**Keywords:** Contact surveillance, Ebola virus disease, Global health crisis, Infection control, Personal protective equipment, Preparedness, Psychological consequences

## Abstract

**Background:**

The current Ebola Virus Disease (EVD) epidemic has ravaged the social fabric of three West African countries and affected people worldwide. We report key themes from an agenda-setting, multi-disciplinary roundtable convened to examine experiences and implications for health systems in Ghana, a nation without cases but where risk for spread is high and the economic, social and political impact of the impending threat is already felt.

**Discussion:**

Participants’ personal stories and the broader debates to define fundamental issues and opportunities for preparedness focused on three inter-related themes. First, the dangers of the fear response itself were highlighted as a threat to the integrity and continuity of quality care. Second, healthcare workers’ fears were compounded by a demonstrable lack of societal and personal protections for infection prevention and control in communities and healthcare facilities, as evidenced by an ongoing cholera epidemic affecting over 20,000 patients in the capital Accra alone since June 2014. Third, a lack of coherent messaging and direction from leadership seems to have limited coordination and reinforced a level of mistrust in the government’s ability and commitment to mobilize an adequate response. Initial recommendations include urgent investment in the needed supplies and infrastructure for basic, routine infection control in communities and healthcare facilities, provision of assurances with securities for frontline healthcare workers, establishment of a multi-sector, “all-hazards” outbreak surveillance system, and engaging directly with key community groups to co-produce contextually relevant educational messages that will help decrease stigma, fear, and the demoralizing perception that the disease defies remedy or control.

**Summary:**

The EVD epidemic provides an unprecedented opportunity for West African countries not yet affected by EVD cases to make progress on tackling long-standing health systems weaknesses. This roundtable discussion emphasized the urgent need to strengthen capacity for infection control, occupational health and safety, and leadership coordination. Significant commitment is needed to raise standards of hygiene in communities and health facilities, build mechanisms for collaboration across sectors, and engage community stakeholders in creating the needed solutions. It would be both devastating and irresponsible to waste the opportunity.

## Background

### Introduction

The Ebola Virus Disease (EVD) epidemic in West Africa has ravaged the social fabric of three countries (Guinea, Liberia, and Sierra Leone) with a death toll of over 11,400 people and over 21,200 cases as of January 15, 2015 [1]. In August 2014 the WHO declared it a Public Health Emergency of International Concern. Travel-associated cases have now been documented in five additional countries, and effects are being felt worldwide.

Efforts to understand this global health crisis and the calls to action have mainly focused on the socioeconomic consequences and erosion of gains in healthcare for people in Guinea, Liberia, and Sierra Leone [2]. Relatively little discussion has examined the impacts and experiences in West African countries that have not yet had EVD cases. These countries share many of the contextual factors that have contributed to the epidemic. While containment efforts at the epicenter are now a clear priority, the crisis presents a clear window of opportunity to address key issues for systems strengthening throughout the sub-region, to prevent further spread and halt the next epidemic should EVD become endemic in the area.

For example Ghana shares the same health systems challenges as the affected countries, including limited financial investment in healthcare, shortage and mal-distribution of healthcare workers, medications and medical supplies, poor health information systems and weak governance [3-5]. These weaknesses create the conditions for endemicity of infectious diseases like Cholera [6] the rising burden of chronic non-communicable diseases like diabetes and hypertension [7,8] and further heightens the lack of preparedness for epidemics like EVD.

In this context, a multidisciplinary group of professionals including economists, anthropologists, mental health professionals, public health professionals, front-line clinicians, population scientists, and historians from Ghana and New York University convened an agenda-setting roundtable on October 23rd at the NYU Accra site in Accra, Ghana. The purpose of the roundtable was threefold. First, to create a forum for mutual support through experience and information sharing. Second, we sought to understand the critical professional and societal needs to support local healthcare capacity and resiliency in the face of this threat. Finally, we aimed to define a means of assisting the preparedness effort.

The roundtable was structured by three moderated discussion sessions, each addressing a broad theme or question (see Table 1). A discussion guide and selection of articles on EVD were sent to all participants in advance. The discussion guide presented a general aim statement and two to five more targeted key questions as the framework for each session. We describe the key themes and analysis that emerged in each of these broad areas.

**Table 1:** **Roundtable agenda themes and questions**

**Session**	**Theme**	**Questions**
1	**Hear the story: Experiences giving patient care during the crisis**	• How difficult has it been to care for patients with unknown infections?
		• How does this experience compare to
		• treating people who might have TB, AIDS or cholera?
2	**Major issues that Ghana is facing in the presence of the Ebola epidemic**	• What are the psychological consequences for communities, patients, families and healthcare workers?
		• How have the policy implications and debates been developed?
		• What are the impacts/risks in communities?
		• What have the operational challenges been for developing preparedness at the level of health facilities?
3	**Looking forward: Panel discussion**	• Can increased access to personal protective equipment transform the infection control culture and improve safety?
		• How can we assist professionals to allow them to retain their professional ethics in the midst of this uncertain hazard and risk?
		• Would the crisis have an impact on brain drain, and what can be done?
		• What can be done to enhance preparedness for the population at large?
		• What can be done to enhance resiliency?

### The stories: experiences giving patient care during the crisis

This session asked “How difficult has it been to care for patients with unknown infections?” Participants offered their personal stories as a basis for discussion. Many of their experiences were strikingly similar to reports coming from the directly affected countries, illustrating how a reasonable yet crippling fear of EVD threatens to erode the esprit de corps and confidence of frontline healthcare workers in the face of this threat to health and health systems.

For example, an emergency department public health nurse noted that healthcare workers already operate daily under high-risk conditions despite their fears, knowing that standards of protection are inadequate. Gloves are never enough, supply of running water inconsistent, and face masks or gowns largely unavailable. Patients often do not disclose highly stigmatized diseases such as TB and HIV, even when the appropriate questions are asked making it difficult to screen for risks. She described how colleagues were reluctant to go near patients with fever during the H1N1 pandemic, and many now say “they will flee if Ebola comes”.

A family physician described facing the “double barrel” threat of EVD during an ongoing cholera outbreak that has affected over 20,000 patients in Accra alone since June 2014, explaining how the public health failure alongside the risks and challenges of managing cases with inadequate staff, space, stuff, and systems has increased fear and insecurity about the ability to manage EVD. Importantly, the cholera experiences included notable success stories of hospital and community mobilization, for example the intervention of Accra’s Mayor to rapidly remodel an existing carport into a new cholera ward.

An internist recounted his experience caring for a patient with EVD-like symptoms. His team had just learned of the death from EVD of a close friend and colleague, who returned to Sierra Leone around the start of the epidemic after training in Ghana. The patient was already admitted onto a general ward when suspicion for EVD was raised. Staff panicked and fled the ward delaying the patient’s care and interrupting care for others on the ward. Feeling grossly underprepared, the doctors threatened strike action. Hospital leadership rapidly acknowledged the concerns in face-to-face meetings, and this was sufficient to avert the strike. However, the situation remained fragile for months as slow progress was made to increase distribution of PPE and develop adequately resourced isolation facilities.

A clinical psychologist underscored the level of inner conflict and threat to professionalism, recounting the experience of her patient who recently gave birth. During delivery, her body fluids splashed onto the midwife, who reacted with horror and a stream of verbal abuse. When the patient later offered payment, the midwife declined the money explaining that she must have caused the young mother such distress by her response.

The institutional contact at the reference lab used for EVD testing described a request for specimen analysis on a patient who was a Nigerian resident in Ghana and had not travelled for several years. He noted that most requests for EVD testing had no associated travel or contact history, and that policy should be refined given the high cost at GHC750 (around 240USD). To provide context, at the time of the Roundtable proceedings, approximately 70 EVD tests had been conducted; to date 150 EVD tests have been conducted. None of the tests have been positive.

### Major issues Ghana is facing in the presence of the Ebola epidemic

This session sought to understand the critical needs from broader existential, organizational, and public health perspectives. Key questions probed the psychological consequences, the impacts/risks in communities, and the operational challenges for preparedness in health facilities. The major discussion themes that emerged cut across different levels of societal response, from the basic unit of the family to communities, health centers, and national strategic efforts.

First, it was clear that widespread fear in relation to perceptions of risk, uncertainty, and the capacity or commitment of leadership to act has been a powerful and prominent force. The fear response affects healthcare workers’ confidence and commitment to professionalism, leads to misuse of scarce healthcare resources such as testing, erodes the trust in the healthcare system, and undermines the needed behavioral and organizational strategies. As one participant suggested, “We’ve demonized this disease, and we need to stop it.”

It was equally clear that many of these fears are not unreasonable given the mismatch between reality and expectations of healthcare delivery. In light of the demonstrable lack of personal and societal protections evidenced by the current cholera outbreak, and chronic deficiencies in the space, systems, staff, and stuff to meet routine healthcare needs, the potential to meet added demands for EVD preparation are widely perceived as unrealistic. Healthcare workers at most institutions in Ghana do not receive workers’ compensation, disability or life insurance, risk allowances, or supplemental health insurance. The threat of EVD increased demands for employers to provide these, and personal advocacy may account for some of the frontline responses.

Similarly, the method and feasibility of a robust contact tracing and monitoring system is unclear. In Ghana, a lack of unique address coordinates for most residences and businesses poses a major threat for ambulance systems to respond, and makes contact tracing difficult. Street names have now been introduced in the larger cities, but finding an individual location is still largely dependent on social coordinates rather than traditional house numbering [9]. Most patients are transported to hospital in taxis, colloquially dubbed “cabulances”, which may also become a significant challenge for infection control.

A third related theme was mistrust in leadership’s ability and commitment to mobilize. Lack of shared knowledge base and vision across professional sectors and societal groups reinforces a disconnect between the myths, science, policies and actual practices for EVD screening and infection control. Heavy politicization of EVD response activities and a lack of coherent messaging and direction from national, local, and institutional leadership paralyzed coordination efforts for months, further undermining confidence in an already fragile system [10]. The mitigation is represented in some areas by intense efforts in team training and acquisition of additional PPE, however much more of this work is needed to achieve capacity, and to establish confidence in an organized response.

Finally, the discussion explored perceived impacts of the EVD threat in Ghana beyond the psychosocial consequences of fear and efforts to prepare. The Ghana government has neither closed borders nor banned intra-regional travel from affected areas, as many advocated. However, tourism and business travel to Accra was markedly reduced in the third and fourth quarters of 2014, when several major international conferences and sports events were cancelled.

## Discussion

### Looking forward - what can build resiliency in the face of this threat to health and healthcare systems?

A final goal for the meeting was to explore opportunities for action, recognizing that an interdisciplinary, multi-level effort is needed to build resiliency, as the United Nations has defined it in the context of disaster risk reduction [11]. EVD transmission can clearly be stopped through rapid mobilization for case and contact finding, early medical care using adequate PPE and environmental controls, and preventive interventions [12]. To do these things requires commitment, confidence, and trust between communities, healthcare workers, and government. Based on the above discussion themes the panel developed the following initial recommendations:

1. Prioritize strengthening infection prevention and control (IPC) in facilities, including reliable water and electricity. This is an urgent priority for both short and long-term investment. In particular, there should be adequate access to and proper training in the use of PPE for all healthcare workers, and that training should be followed up with structured programs for supervision, monitoring and evaluation. IPC is a critical aspect of EVD prevention, and should be particularly important as a focus for systems strengthening given the endemicity of cholera and tuberculosis in Ghana [6]. Investing in this critical competency is equally essential to build staff confidence and trust in leadership’s commitment to quality care and their own occupational health and safety. The risk of an EVD patient escaping detection at triage seems considerable in light of the non-specific nature of symptoms, the experiences in Lagos, Port Harcourt, and Senegal where index patients did not initially disclose their EVD contacts, and the likelihood that - looking to the future - the next outbreak will come unannounced.
2. Provide assurances with securities for frontline healthcare workers. Specifically, healthcare workers should have guaranteed health insurance, workers’ compensation and disability insurance should they contract EVD in the process of screening and providing care to patients. The needs of their families should also be addressed, including options for isolation of healthcare workers outside of the home to protect their families from becoming infected. Stories of abandonment and violence toward frontline healthcare workers in Guinea, Liberia, and Sierra Leone fuel staff insecurities and are a barrier to continued quality care in Ghana as evidenced by the reactions to EVD suspects in various health facilities to date. Conversely, making sure that nurses had their home needs met was an important factor in maintaining health systems integrity in Canada during the SARS pandemic [13].
3. Establish a continuous surveillance system to monitor and document all suspected cases and contacts in the community. This should take advantage of existing local resources such as the National Disaster Management Organization (NADMO), and the epidemiological surveillance programs at University of Ghana School of Public Health in addition to those run though the Ministry of Health and Ghana Health Service. It should be well linked to and coordinated within an active network of multiagency multi-sector community and local government partners capable of responding not only to a projected EVD outbreak, but to regularly recurring outbreaks such as cholera that frequently destabilize health and health systems in Ghana [10].
4. Develop a coordinated and consistent education on EVD transmission and the current epidemic. The educational messages should be co-produced with the community leaders, organizations, work unions, frontline healthcare workers and other community-based organizations. Poor health literacy and information access in communities, and even among healthcare workers, contributes to fear and mistrust. Public health officials in Uganda cite massive public education as a key determinant in their success containing multiple viral hemorrhagic fever outbreaks [14]. Messages should have two very important attributes. First, they should demystify the disease by focusing on the science of what works to control spread. Second, they should highlight the various stories of resiliency and positive case examples such as survivors’ stories and the successful responses in Uganda, Nigeria, and Senegal. This education is critical to combat the erroneous perception that EVD defies control, to decrease fear and stigma, and to encourage care-seeking. However, as studies of risk perception suggest, the communication must be bi-directional and contextual to be effective [15]. We need go to the communities and ask what their members understand, what fears they harbor, what practical challenges and concerns they have to implement the key recommendations, and how they think the issues should be addressed. This is the core aim of co-production. Engaging with local religious and community leaders early and often to develop acceptable solutions in context has been a vital component of successful EVD containment strategies in the past [16-18]. Enlisting the media as allies should also be of critical importance.

## Summary

Historically, the ability of African nations to manage infectious disease outbreaks has largely been understated and often dependent on external ‘thinkers’ and aid. Clearly massive outside assistance is needed to contain the current EVD epidemic, yet the critical roles of local leadership and communities cannot be overemphasized. The current crisis provides an unprecedented opportunity for West African countries not yet affected to make advances on creating culturally and contextually relevant solutions to build resiliency and strengthen health systems for outbreak control. This will require significant investment and support to raise standards of hygiene and protection in communities and healthcare facilities, to build mechanisms for coordination and collaboration across sectors, and to enlist the people on the frontline - in families, communities, and healthcare facilities - as allies in the process.

Fear is only a weakness if it turns to panic. If harnessed appropriately, fear can be a stimulus for wide-ranging changes in health system strengthening. A case in point is the successful response to Ebola outbreak in Nigeria [19]. Because forewarned is forearmed, it would be both devastating and irresponsible to continue “business as usual” and waste the opportunity.

## Abbreviations

EVD: Ebola virus disease
HIV: Human immunodeficiency virus
IPC: Infection prevention and control
NADMO: National Disaster Management Organization
NYU: New York University
NYU CTED: New York University Center for Technology and Economic Development
TB: Tuberculosis
